# Monocyte Differentiation towards Protumor Activity Does Not Correlate with M1 or M2 Phenotypes

**DOI:** 10.1155/2016/6031486

**Published:** 2016-06-08

**Authors:** G. Karina Chimal-Ramírez, Nancy Adriana Espinoza-Sánchez, Luis Chávez-Sánchez, Lourdes Arriaga-Pizano, Ezequiel M. Fuentes-Pananá

**Affiliations:** ^1^Unidad de Investigación en Virología y Cáncer, Hospital Infantil de México Federico Gómez, Dr. Márquez 162, Colonia Doctores, 06720 Ciudad de México, DF, Mexico; ^2^Programa de Doctorado en Ciencias Biomédicas, Universidad Nacional Autónoma de México (UNAM), 04510 Ciudad de México, DF, Mexico; ^3^UIM en Inmunología, Hospital de Pediatría, CMN Siglo XXI, IMSS, 06720 Ciudad de México, DF, Mexico; ^4^UIM en Inmunoquímica, Hospital de Especialidades, CMN Siglo XXI, IMSS, 06720 Ciudad de México, DF, Mexico

## Abstract

Macrophages facilitate breast cancer progression. Macrophages were initially classified as M1 or M2 based on their distinct metabolic programs and then expanded to include antitumoral (M1) and protumoral (M2) activities. However, it is still uncertain what markers define the pro- and antitumoral phenotypes and what conditions lead to their formation. In this study, monocytic cell lines and primary monocytes were subjected to commonly reported protocols of M1/M2 polarization and conditions known to engage monocytes into protumoral functions. The results showed that only IDO enzyme and CD86 M1 markers were upregulated correlating with M1 polarization. TNF-*α*, CCR7, IL-10, arginase I, CD36, and CD163 were expressed indistinguishably from M1 or M2 polarization. Similarly, protumoral engaging resulted in upregulation of both M1 and M2 markers, with conditioned media from the most aggressive breast cancer cell line promoting the greatest changes. In spite of the mixed phenotype, M1-polarized macrophages exhibited the highest expression/secretion of inflammatory mediators, many of which have previously been associated with breast cancer aggressiveness. These data argue that although the existence of protumoral macrophages is unquestionable, their associated phenotypes and the precise conditions driving their formation are still unclear, and those conditions may need both M1 and M2 stimuli.

## 1. Introduction

The study of macrophage behavior in both pathological and normal conditions has shown the versatility of these cells beyond their basic well-known immune effector functions. Although macrophages were first understood as potent removers of invading pathogens through their phagocytic and antigen presenting activities, these cells are most often engaged in clearing of aged cells in nonpathologic conditions. Moreover, macrophages express a battery of bioactive molecules that promote tissue remodeling/healing, support cell proliferation and angiogenesis, and mediate immunosuppression under certain microenvironmental conditions [1, 2].

Mills and colleagues in 2000 [3] were the first to separate macrophages into M1 and M2 subclasses based on their antagonistic metabolic programs and to mirror the T cell literature. This group noticed that macrophages derived from mouse strains with preferential Th1-responses (e.g., C57BL/6 or B10D2) yielded larger quantities of end-products of the induced nitric oxide synthase (iNOS), while macrophages derived from Th2-strains (e.g., BALB/c or DBA/2) preferentially synthesized ornithine, a product of arginase. Macrophages were then referred to as M1 or M2 to relate them with their associated Th1 or Th2 immune responses. Mills and colleagues pointed out very clearly that their classification was mainly useful to explain their findings and that the M1 and M2 phenotypes might not correspond to “clonally separable cells.” Still, those observations led to the conclusion that there may be macrophages in a spectrum of different phenotypes and functions [4].

M1 and M2 macrophages have also been identified as pro- and anti-inflammatory macrophages, respectively. Moreover, according to the aforementioned functional profiles, M1 proinflammatory macrophages are considered to have antitumoral responses, while macrophages that display anti-inflammatory responses are thought to support protumoral functions and are included in the M2 classification, thus evidencing how widely the M1 and M2 terms have been used. Particularly in cancer, there is conflicting evidence of the role that macrophages play during cancer initiation and progression. On one hand macrophages are efficient killers of tumor cells, and on the other, increasing evidence places macrophages as powerful drivers of oncogenesis and promotion towards aggressive tumors. Colony-stimulating factor-1 (CSF-1) has been well documented as a powerful regulator of macrophage proliferation, differentiation, and survival [5], and high levels of CSF-1 and its receptor were later found to point out to human cancers with poor prognosis [6, 7]. In the pioneer study by Lin et al., impediment of peripheral monocytes arrival to the tumor stroma after CSF-1 knockout reduced tumor growth and delayed invasion and metastasis [8]. It was later found in humans that high density of macrophages in the tumor stroma significantly correlates with cancers of poor prognosis, and in histological sections of invasive tumors macrophages were preferentially located in areas of active protease secretion and increased basement membrane degradation [9–13]. Today, it is well accepted that tumor associated macrophages (TAMs) can be an important component of the pathogenic responses that drive the most aggressive tumors. TAMs favor invasion, angiogenesis, intravasation, extravasation, and metastasis through secretion of interleukins, chemokines, growth factors, and proteases [14–16]. Because these responses are more attuned to tissue remodeling or wound healing functions, TAMs have generally been described as M2 macrophages. Nevertheless, macrophages polarized in tumor stroma-like experimental conditions coexpress M1 and M2 markers [1, 11, 17, 18]. Thus, there is still discrepancy regarding the markers to define the TAMs profile.

We recently found that monocytes cocultured in 3-dimensional (3D) conditions with breast cancer cell lines are instructed to switch gene expression to a pattern more attuned to tumor-promoting activities; for instance, we observed increased expression of the COX-2 inflammatory mediator and its metabolite prostaglandin E2 and increased levels of metalloproteinases that correlated with increased collagen degradation [19]. In this study, we have tested the most commonly used protocols to polarize macrophages into M1 and M2 subtypes together with culturing them with conditioned media obtained from aggressive and nonaggressive breast cancer cell lines. Macrophage subtype classification was then addressed phenotypically, as well as through their phagocytic activity and profile of cytokine expression.

## 2. Material and Methods

### 2.1. Cell Culture

All cell lines were obtained from the American Type Culture Collection (ATCC, Manassas, VA, USA) and culture media and supplements from Gibco BRL Life Technologies (Grand Island, NY, USA) unless specified. Human monocytic cell lines THP-1 and U937 were cultured in RPMI 1640 medium supplemented with 10% fetal bovine serum (FBS), 100 U/mL penicillin, and 100 U/mL streptomycin, at 37°C in 5% CO_2_. Cell stocks were frozen at a density of 2-3 million cells per mL, with RPMI 1640 medium supplemented with 20% FBS and 10% dimethylsulfoxide (DMSO, Sigma Life Science, St. Louis, MO, USA). Breast cancer cells MCF-7 and MDA-MB-231 were cultured in DMEM/F12 medium supplemented with 10% FBS, 100 U/mL penicillin, and 100 U/mL streptomycin, at 37°C in 5% CO_2_.

### 2.2. Isolation of Peripheral Blood Primary Monocytes

Peripheral blood mononuclear cells (PBMCs) were isolated according to the following protocol: 35–40 mL of blood of at least two healthy volunteers was extracted, diluted in a 1 : 3 proportion with sterile Phosphate Buffered Saline (PBS, Gibco BRL Life Technologies, Grand Island, NY, USA), and subjected to density gradient centrifugation with Histopaque®-1077 (Sigma Aldrich Inc., St. Louis, MO, USA) per 30 minutes at 2000 rpm. PBMCs were then carefully retrieved from the gradient and washed 3 times with PBS, each time followed by slower centrifugation (1500, 1250, and 1000 rpm). To obtain the monocyte-enriched fraction, PBMCs were subjected to negative selection with the Monocyte Isolation Kit II Human (Miltenyi Biotec Inc., Auburn, CA, USA) following the manufacturer's recommendations as we briefly describe next. PBMCs were washed once with diluted 1 : 20 MACS BSA Stock Solution (Miltenyi Biotec Inc., Auburn, CA, USA), counted, and adjusted to a density of 10^7^ cells per 30 *μ*L of buffered solution; 10 *μ*L of FcR blocking reagent and 10 *μ*L of monocyte biotin-antibody cocktail were then added for every 10^7^ cells to be labeled; cells were mixed and incubated for 15 minutes at 4°C. An additional 30 *μ*L of buffered solution was added plus 20 *μ*L of antibiotin microbeads for every 10^7^ cells to be labeled; cells were mixed and incubated for 20 minutes at 4°C. Cells were then washed once with buffered solution, centrifuged at 1500 rpm for 5 minutes, and resuspended in 1–1.5 mL of buffered solution for magnetic separation. Suspension of cells was passed through a prerinsed LS column (Miltenyi Biotec Inc., Auburn, CA, USA) and 6-7 mL of buffered solution was added. Entire effluent was collected in a conical 15 mL tube identified as the monocyte-enriched fraction. Collected cells were counted and if not cultured immediately they were frozen at a density of 2 × 10^6^ with DMEM/F12 medium supplemented with 50% FBS and 10% DMSO at −80°C. Frozen monocytes were thawed and cultured after no more than 2 months of freezing. Cultures were done in DMEM/F12 medium supplemented with 6% FBS, 100 U/mL penicillin, and 100 U/mL streptomycin, at 37°C in 5% CO_2_. Each set of experiments was performed utilizing primary monocytes of at least two different donors, and the number and phenotype of purified monocytes were very homogeneous.

### 2.3. Activation of Monocytes

According to the literature consulted, each source of monocytes used as a model of activation (THP-1, U937, and primary monocytes) has different requirements to achieve an activated state, including different incubation periods and cytokines; therefore treatments were adapted for each case as follows. THP-1 cells were subjected to different stimulation treatments in RPMI 1640 medium supplemented with a low FBS concentration (2%), at a density of 2 × 10^5^ cells per well in 24-well flat-bottom culture plates [17, 20–22]. Activation treatments consisted of (1) no stimulation control (mock); (2) phorbol 12-myristate 13-acetate (PMA, Sigma Aldrich Inc., St. Louis, MO, USA) 30 ng/mL for 4 h (PMA-only control); (3) pretreatment with PMA 30 ng/mL for 4 h, followed by LPS (Sigma Aldrich Inc., St. Louis, MO, USA) 10 ng/mL and INF-*γ* (R&D Systems Inc., Minneapolis, MN, USA) 5 ng/mL for 72 h (condition favoring M1 polarization); (4) pretreatment with PMA 30 ng/mL for 4 h, followed by IL-4 (Sigma Aldrich Inc., St. Louis, MO, USA) 25 ng/mL and IL-13 (Sigma Aldrich Inc., St. Louis, MO, USA) 25 ng/mL for 72 h (condition favoring M2 polarization); and (5) IL-4 25 ng/mL and IL-13 25 ng/mL for 72 h (condition favoring M2 polarization without PMA).

The activation of U937 cells highly resembles the activation conditions of THP-1 cells; however incubation times differ. Also, for these monocytes an additional treatment with M-CSF was included based on the references consulted. Although M-CSF has also been used to stimulate THP-1 cells, it has not been specified whether it achieved an M2 polarization [23–25]. U937 cells were also treated in RPMI 1640 medium supplemented with 2% FBS, at a density of 2 × 10^5^ cells per well in 24-well flat-bottom culture plates. Activation treatments consisted of (1) no stimulation control (mock); (2) PMA 20 ng/mL for 48 h (PMA-only control); (3) pretreatment with PMA 20 ng/mL for 48 h, followed by LPS 50 ng/mL and INF-*γ* 10 ng/mL for 96 h (condition favoring M1 polarization); (4) pretreatment with PMA 20 ng/mL for 48 h, followed by IL-4 25 ng/mL and IL-13 25 ng/mL for 96 h (condition favoring M2 polarization (M2-A)); (5) pretreatment with PMA 20 ng/mL for 48 h followed by M-CSF 20 ng/mL for 72 h (condition favoring M2 polarization (M2-B)); and (6) IL-4 25 ng/mL and IL-13 25 ng/mL for 96 h (condition favoring M2 polarization without PMA (M2-C)) [26–29].

For primary monocyte activation, cells were treated in DMEM/F12 medium supplemented with 6% FBS, at a density of 2 × 10^5^ cells per well in 24-well flat-bottom culture plates in the following conditions: (1) no stimulation control (mock); (2) pretreatment with GM-CSF (PeproTech Inc., Rocky Hill, NJ, USA) 100 ng/mL for 6 days followed by LPS 100 ng/mL and INF-*γ* 25 ng/mL for 48 h (condition favoring M1 polarization); and (3) pretreatment with M-CSF (PeproTech Inc., Rocky Hill, NJ, USA) 100 ng/mL for 6 days followed by IL-4 25 ng/mL and IL-13 25 ng/mL for 48 h (condition favoring M2 polarization) [17, 30, 31]. Treated cells were carefully harvested by rinsing with PBS and mild trypsinization when needed.

### 2.4. Monocyte Treatment with Conditioned Media Obtained from Breast Cancer Cell Lines

THP-1, U937, and primary monocytes were plated at a density of 2 × 10^5^ cells/mL/well in 24-well flat-bottom culture plates in a 1 : 1 mix of RPMI 1640 medium (2% FBS) and either MCF-7 or MDA-MB-231 supernatant. A control with a 1 : 1 mix of RPMI 1640 medium (2% FBS) and no supplemented DMEM/12 was included. After incubation for 5 days (with one replacement of correspondent media after 48 h) cells were harvested as indicated above.

### 2.5. Harvest of Cell Culture Supernatants

Two × 10^6^ MCF-7 and MDA-MB-231 cells were plated in 182 cm^2^ cell culture flasks in standard supplemented medium. When cultures reached 80% confluence supernatants were discarded, cells were rinsed with PBS, and then 20 mL of DMEM/F12 without FBS was added. Supernatants were harvested after incubation for an additional 48 h, centrifuged at 1500 rpm/5 min, aliquoted, and stored at −20°C until use. Supernatants from treated monocytes were also collected for analysis of cytokine secretion. For this, supernatants were collected after finishing treatment and centrifuged at 1500 rpm/5 min, aliquoted, and stored at −20°C until use.

### 2.6. Flow Cytometry

Initial characterization of monocytes: all three types of monocytes were washed twice with washing buffer (3% FBS in PBS) and incubated in 100 *μ*L of blocking solution (50% FBS in PBS) at room temperature (RT) for 15 minutes. After incubation in blocking solution 50 *μ*L of a 1 : 50 dilution in washing buffer of antibodies was added (for panel 1 staining: mouse anti-human anti-CD34-allophycocyanin (APC), mouse anti-human anti-CD11b-phycoerythrin (PE), and mouse anti-human anti-CD14-fluorescein isothiocyanate (FITC); for panel 2 staining: mouse anti-human anti-CD64-APC, mouse anti-human anti-CD68-PE, and mouse anti-human anti-CD16-FITC; all antibodies from BioLegend, San Diego, CA, USA) and incubated at RT for 30 minutes. Cells were then washed once with 1 mL of washing buffer, centrifuged, and resuspended in 150 *μ*L of PBS for acquisition in BD FACS CANTO Flow Cytometer and analyzed with the FlowJo V10 Software.

Phenotyping of polarized monocytes: monocytes were phenotyped by flow cytometry after the various treatments as follows. Monocytes were washed twice with washing buffer (1% FBS 0.01% NaN_3_ PBS) and incubated in 100 *μ*L of blocking solution (25% FBS 15% human serum PBS) at 4°C for 20 minutes. After incubation in the blocking solution 100 *μ*L of a 1 : 100 dilution in blocking solution of antibodies was added (for panel 1 staining: mouse anti-human anti-CD86-PE and anti-CD163-peridinin­chlorophyll protein complex (PerCP); for panel 2 staining: rat anti-human anti-CD36-fluorescein and mouse anti-human anti-CCR7-PerCP; all antibodies from R&D Systems) and incubated at 4°C for 30 minutes. Cells were then washed once with 1 mL of washing buffer, fixed, and permeated with 200 *μ*L of Fixation/Permeabilization Solution (BD Biosciences, San Jose, CA, USA) and incubated at 4°C for 20 minutes. Cells were then washed with the BD Perm/Wash*™* buffer (BD Biosciences, San Jose, CA, USA), centrifuged, and resuspended in 100 *μ*L of Perm/Wash buffer. 100 *μ*L of a 1 : 100 dilution in Perm/Wash buffer of antibodies against intracellular antigens was added (for panel 1 staining: mouse anti-human anti-TNF-*α*-fluorescein and anti-IL-10-APC; for panel 2 staining: mouse anti-human anti-arginase I-PE and mouse anti-human anti-indoleamine-pyrrole 2,3-dioxygenase- (IDO-) APC; all antibodies from R&D Systems Inc., Minneapolis, MN, USA) and incubated at 4°C for 30 minutes. After incubation cells were washed with 1 mL of Perm/Wash buffer, centrifuged, and resuspended in 150 *μ*L of PBS for acquisition in BD FACS CANTO Flow Cytometer and analyzed with the FlowJo V10 Software.

### 2.7. Analysis of Cytokine Profiles

To determine the cytokine profiles present in monocytes supernatants after the various polarization treatments, concentrations (pg/mL) of G-CSF (granulocyte-colony-stimulating factor), GM-CSF (granulocyte-macrophage-colony-stimulating factor), IL-1*β* (interleukin-1 beta), IL-8 (interleukin-8), IL-12p70 (interleukin-12p70), INF-*α*2 (interferon-alpha 2), MCP-1 (monocyte chemoattractant protein-1, also known as CCL2), EGF (Epidermal Growth Factor), VEGF (Vascular Endothelial Growth Factor), and RANTES (regulated on activation, normal T cell expressed and secreted, also known as chemokine CCL5) were measured with the MILLIPLEX HCYTOMAG-60 K Kit (EMD Millipore Corporation, Billerica, MA, USA) following the manufacturer's recommended procedure. Briefly, in each well of a 96-well flat-bottom culture plate 25 *μ*L of assay buffer was mixed with 25 *μ*L of supernatants or controls and 25 *μ*L of the detection microbeads cocktail. The mixture was incubated at 4°C overnight with orbital agitation. Wells were then washed twice with washing buffer (included in the kit) and 25 *μ*L of the detection antibodies mix was added to each well and the plate was incubated at RT with orbital agitation for 1 h. After incubation, 25 *μ*L of streptavidin-PE was added to each well followed by 30 more minutes of incubation at RT with orbital agitation. The wells were then washed twice with washing buffer and 150 *μ*L of PBS was added to each well to proceed with the analysis in Luminex MAGPIX multiplexing instrument and the analysis of data was performed in the xPONENT® Software.

### 2.8. Phagocytosis Assay

Monocyte suspensions after the various treatments were counted and 1 × 10^5^ cells were plated per well of a 96-well flat-bottom culture plate in 100 *μ*L of medium. Cells were allowed to settle at the bottom of the wells, the supernatant was carefully aspirated from each well, and 100 *μ*L of fluorescein-labeled* Escherichia coli* K-12 BioParticles (Vybrant Phagocytosis Assay Kit, Molecular Probes Inc., Eugene, OR, USA) was added; monocytes were then incubated at 37°C in 5% CO_2_ for 2 h. After incubation the BioParticles were carefully aspirated from each well, 100 *μ*L of trypan blue (Vybrant Phagocytosis Assay Kit, Molecular Probes Inc., Eugene, OR, USA) was added to each well, and the plate was incubated for 1 minute at RT; trypan blue was then aspirated and fluorescence present within the cells was detected in an* Ascent* fluorometer with an excitation wavelength of 480 nm and emission of 520 nm. A series of at least 3 blank wells were included to subtract background fluorescence to the sample's emissions. Phagocytic activity was expressed as mean fluorescence intensity of at least five technical replicates after subtraction of the average fluorescence intensity of the group of blank wells. Three independent experiments were performed.

### 2.9. Statistical Analysis

Statistical comparison of values from the different conditions tested was performed with the GraphPad Prism 5 Software, using one-way analysis of variance (ANOVA) test and Tukey's posttest to compare all pairs of data columns. Significance ≤0.05 was indicated with *∗*, ≤0.01 was indicated with *∗∗*, and ≤0.0005 was indicated with *∗∗∗*.

## 3. Results

### 3.1. Phenotypic Characterization of Monocytic Cell Lines and Primary Monocytes

**Figure 1:**
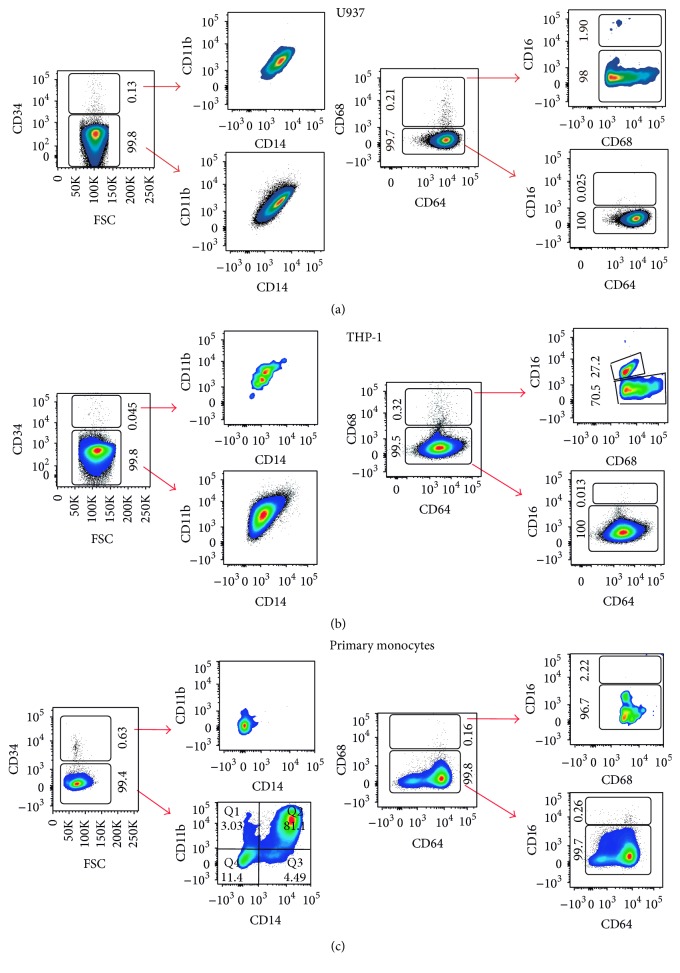
Phenotypic characterization of monocytic cell lines and primary monocytes. Dot blots representative of the flow cytometry analysis. Depicted are population percentages based on two marker panels: CD34, CD11b, and CD14 (left) and CD68, CD64, and CD16 (right) markers for (a) U937 cells, (b) THP-1 cells, and (c) primary monocytes. One donor is used as an example for primary monocytes although similar results were obtained with different donors and with different isolation batches. The phenotype found in most of the cells analyzed is basically the same for all three types of monocytes: CD34^neg^, CD11b^pos^, CD14^pos^, CD68^neg^, CD64^pos^, and CD16^neg^, which denotes a classical inactivated monocyte pattern.

U937 and THP-1 are myeloid cells derived from patients with a histiocytic lymphoma and monocytic leukemia, respectively, which are often used to study monocyte differentiation. The phenotypes of both monocytic cell lines together with primary monocytes derived from peripheral blood of healthy donors were characterized by flow cytometry to know their stage of differentiation. U937 cells were almost entirely CD34^neg^ (99.8%) CD11b^pos^  CD14^pos^, which outlines their myeloid lineage, although already compromised to monocytes. 99.7% of these cells were also CD64^pos^  CD68^neg^  CD16^neg^, which shows that U937 cells are mostly in an undifferentiated inactive monocyte state (Figure 1(a)). THP-1 cells were very similar; >99% of the population consisted of CD34^neg^  CD11b^pos^  CD14^pos^  CD64^pos^  CD68^neg^  CD16^neg^. These features along with their effortless maintenance in culture make U937 and THP-1 cells a suitable* in vitro* experimental model for macrophage differentiation and activation. One slight difference found was that THP-1 cells had a very small fraction (about 0.1% of the total population) of CD68^pos^  CD16^pos^ cells, which indicates the presence of activated monocytes (Figure 1(b)).  Figure 1(c) shows a representative characterization of one of the isolates of primary monocytes. There was more heterogeneity in the cell population found in primary monocytes; 99.4% of these cells were CD34^neg^, from which 81.1% were CD11b^pos^  CD14^pos^ myeloid cells. 99.8% of primary monocytes were also CD68^neg^  CD64^pos^  CD16^neg^ and the small fraction (0.16%) of CD68^pos^ cells was also CD16^neg^. This profile denotes also a classical monocytic profile.

### 3.2. Morphological Characterization of M1- or M2-Polarized Monocytes

**Figure 2:**
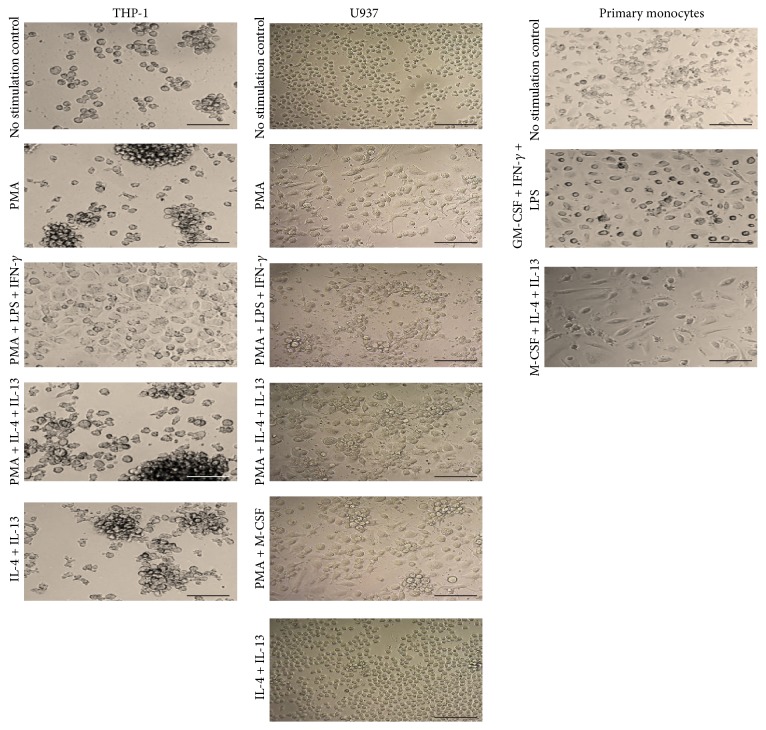
Morphological characterization of M1- or M2-polarized monocytes. Morphology observed for THP-1, U937, and primary monocytes under the indicated treatments. Images were taken with Motic Microscope at 200x magnification; scale bar indicates 50 *μ*m. The most relevant features of differentiation were adherence, stronger in M1-polarizing treatments, and several cell shape changes in most cases presenting mixed shapes (round and spindle-like and with several membrane projections) and cell sizes.

Differentiation from monocyte to macrophage is accompanied by cell morphological changes.  Figure 2 shows that THP-1 and U937 cells had very similar basal morphology and exhibited very similar changes after culture in M1- or M2-polarizing conditions. Both cell lines presented a rounded nonadherent basal morphology, which upon treatment with PMA alone or with M2-polarizing conditions became elongated with some cells displaying adherence. These changes were more sizable after treatment with M1-polarizing conditions. Cell aggregates were also observed in the THP-1 cells, which became larger after treatment with PMA alone or with M2-polarizing conditions. Primary monocytes were characterized by a mixed morphology in conditions of no stimulation; cells were observed in variable sizes and shapes with some of them showing spindle-like forms and membrane projections. Cells were also moderately adherent and became highly adherent after treatment with GM-CSF + INF-*γ* + LPS (M1 conditions), showing also evident nuclei. In contrast, primary monocytes treated with M-CSF + IL-4 + IL-13 (M2 conditions) were mostly spindle-like shaped and less adherent.

### 3.3. Characterization of M1- and M2-Related Phenotypes

**Figure 3:**
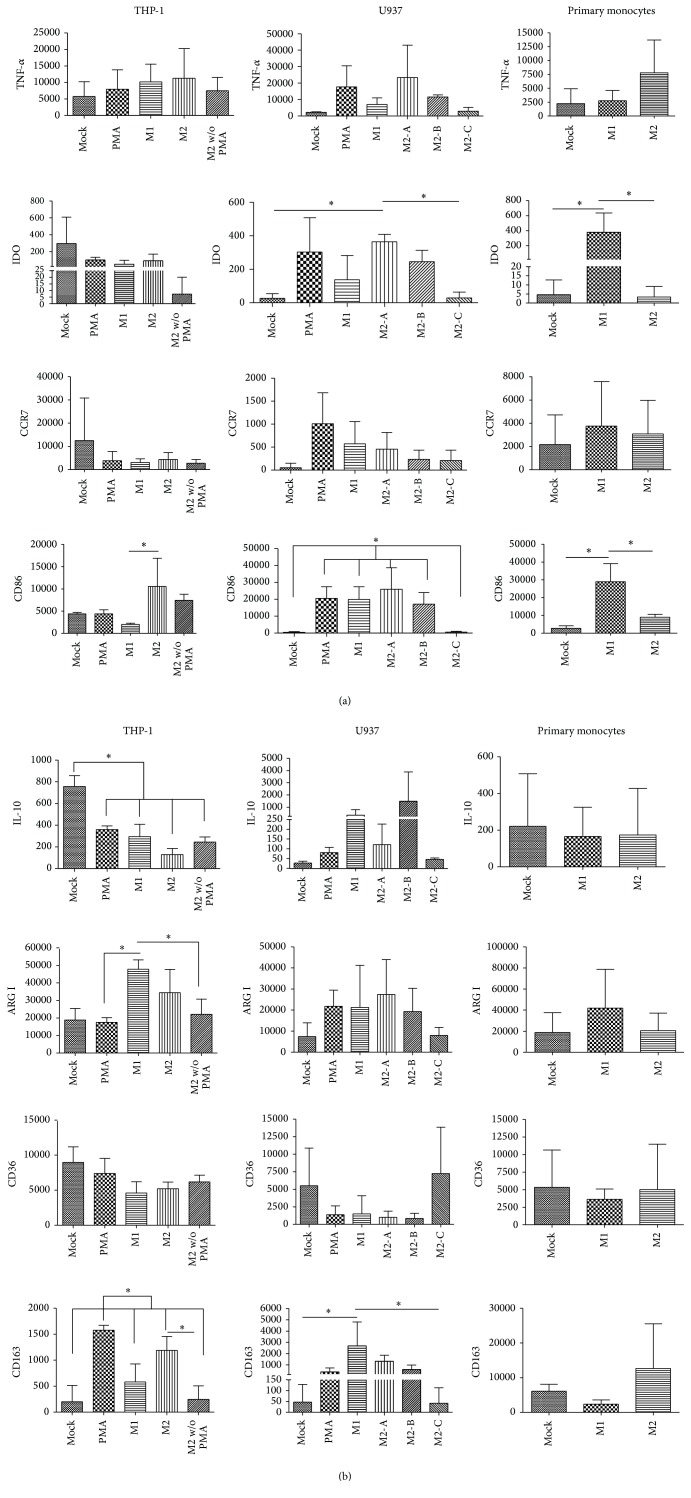
Characterization of M1- and M2-related phenotypes. Median fluorescence intensity corresponding to (a) M1-related markers TNF-*α*, IDO enzyme, CCR7, and CD86 and (b) M2-related markers IL-10, arginase I, CD36, and CD163, found in cells harvested from all different polarizing treatments and controls and analyzed by flow cytometry. For THP-1, mock: no stimulated cells; PMA: PMA-only control; M1: PMA + LPS + INF-*γ*; M2: PMA + IL-4 + IL-13; and M2 w/o PMA: IL-4 + IL-13. For U937, mock: no stimulated cells; PMA: PMA-only control; M1: PMA + LPS + INF-*γ*; M2-A: PMA + IL-4 + IL-13; M2-B: PMA + M-CSF; and M2-C: IL-4 + IL-13. For primary monocytes, mock: no stimulated cells; M1: GM-CSF + LPS + INF-*γ*; and M2: M-CSF + IL-4 + IL-13. Asterisks and bars denote statistical significance between conditions. Significant differences (*p* ≤ 0.05) found were as follows: in THP-1 cells CD86, M1 versus M2; IL-10, mock versus all the rest of the conditions; arginase I, M1 versus PMA and versus M2 without PMA; CD163, PMA versus all the rest of the conditions and M2 versus M2 without PMA. In U937 cells IDO, M2-A versus mock and versus M2-C; CD86, (PMA, M1, M2-A, and M2-B) versus mock and M2-C; CD163, M1 versus mock and versus M2-C. In primary monocytes IDO and CD86 were significantly different in M1 versus mock and versus M2.

Several markers previously reported as being M1- or M2-related were analyzed in untreated or polarized cells by flow cytometry. For M1 the markers considered were TNF-*α*, IDO enzyme, CCR7, and CD86 and for M2 they were IL-10, arginase I enzyme, CD36, and CD163. Cells were analyzed after polarization by flow cytometry and Figures 3(a) and 3(b) show the results expressed as the median fluorescence intensity (MFI). Contrary to what is expected, we found that the levels of all markers of M1 or M2 were very variable impeding assignment of a clear M1 or M2 phenotype. Also, the basal expression levels were very variable between cells, in spite of the highly similar differentiation stage. For instance, THP-1 cells express more of the M1 markers than U937 and primary monocytes and of IL-10 and CD36 M2 markers, while primary monocytes express higher levels of arginase I and CD163. Interestingly, even the basal levels of expression were highly variable within one type of cell suggesting very responsive cellular stages. In agreement with that, PMA treatment alone often resulted in levels of M1 or M2 markers as high as after M1- or M2-polarizing conditions.

Analyses of M1 markers (Figure 3(a)) showed that only primary monocytes displayed a correlative significant increased expression of M1 markers IDO and CD86 after polarization with M1 conditions. CD86 behaved contrary to what is expected in THP-1 cells, since it was higher after treatment with PMA + IL-4 + IL-13 M2-polarizing conditions. In U937 cells only the IL-4 + IL-13 treatment (M2-C) did not result in upregulation of CD86. Since M2-C is the only condition that does not include PMA, this result suggests that PMA is a potent inductor of CD86 in these cells. IDO was significantly more expressed after PMA + IL-4 + IL-13 (M2-A) treatment than after IL-4 + IL-13 (M2-C), while M1 treatment did not significantly increase IDO expression.

Meanwhile, for M2-related markers, we did not find a* bona fide* marker whose expression varied specifically according to the M2-polarizing conditions (Figure 3(b)). If we had only compared M2 against mock conditions, U937 would have been the cells closer to the expected behavior. In these cells IL-10, arginase I, and CD163 were higher after M2 conditions, although nonsignificant. In THP-1 cells, arginase I and CD163 showed a nonsignificant increased expression between M2-polarization and mock. However, M2 markers were also upregulated after treatment with M1-polarizing conditions. In U937 cells CD163 was significantly higher in M1 conditions than after IL-4 + IL-13 (M2-C) treatment, and in THP-1 cells IL-10 was significantly higher in mock than in any other condition. Primary monocytes untreated or treated showed very similar levels of M2 markers.

### 3.4. Determination of Phagocytosis

**Figure 4:**
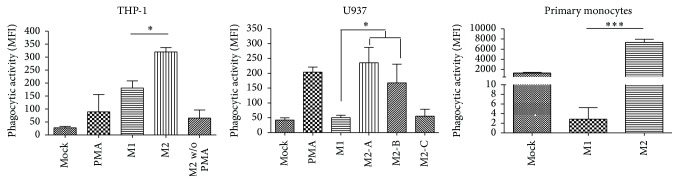
Determination of phagocytosis. Phagocytic activity of THP-1, U937, and primary monocytes expressed as the median fluorescence intensity of fluorescein-labeled* E. coli* K-12 BioParticles engulfed by cells. Mean of three independent experiments is shown. For THP-1, mock: no stimulated cells; PMA: PMA-only control; M1: PMA + LPS + INF-*γ*; M2: PMA + IL-4 + IL-13; and M2 w/o PMA: IL-4 + IL-13. For U937, mock: no stimulated cells; PMA: PMA-only control; M1: PMA + LPS + INF-*γ*; M2-A: PMA + IL-4 + IL-13; M2-B: PMA + M-CSF; and M2-C: IL-4 + IL-13. For primary monocytes, mock: no stimulated cells; M1: GM-CSF + LPS + INF-*γ*; and M2: M-CSF + IL-4 + IL-13. Asterisks and bars denote statistical significance between conditions. Significance ≤0.05 was indicated with *∗*, and ≤0.0005 was indicated with *∗∗∗*. In THP-1 cells and primary monocytes there was statistically significant differences between M1 and M2 conditions and in U937 between M1 and M2-A and M2-B.

In order to better understand the functional activity of M1- and M2-polarized monocytes/macrophages we performed a phagocytosis assay. This assay consisted of measuring the fluorescence intensity of engulfed fluorescein-labeled* E. coli* K-12 BioParticles. Both M1 and M2 macrophages are highly phagocytic although to different substrates; while M1 macrophages are in charge of pathogen clearing, M2 macrophages remove aged or damaged cells. Because the assay was based on* E. coli*, we were expecting an increased activity given by M1-polarized monocytes. Strikingly, we found that all three types of monocytes tested showed the highest phagocytic activity in M2 conditions (Figure 4). In the cases of THP-1 and U937 cells this elevated phagocytic capability matched the conditions where prestimulation with PMA was performed, and in concordance with that, PMA treatment alone also showed a significant increased activity compared to mock cells (Figure 4 only shows the M1-versus-M2 statistical analysis). Although M2-polarized THP-1 and U937 cells without PMA (only IL-4 and IL-13) were poorly phagocytic, this activity does not seem to be exclusively mediated by PMA since M1 polarization also uses PMA. Moreover, M2-polarized primary monocytes, in which PMA was not used, were also highly phagocytic.

### 3.5. Analysis of the Profile of Cytokine Expression

**Figure 5:**
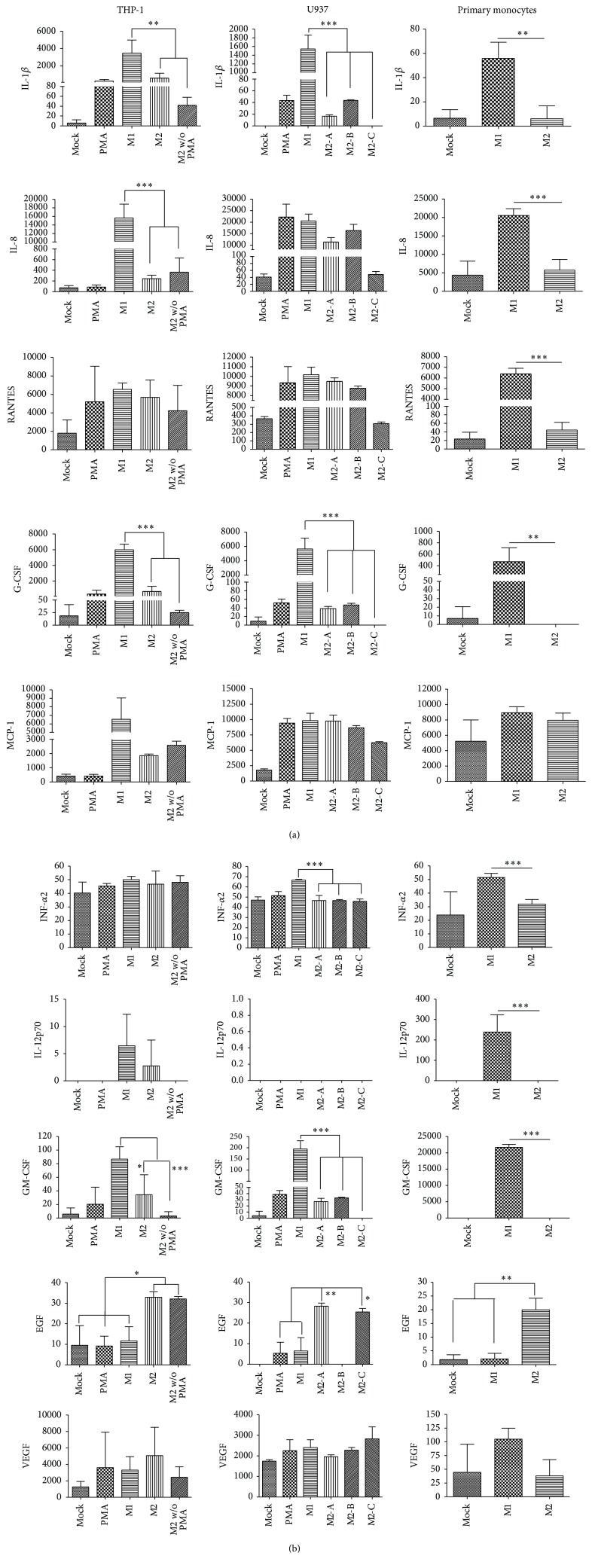
Analysis of the profile of cytokine expression. Concentrations expressed in pg/mL of IL-1*β*, IL-8, RANTES, G-CSF, MCP-1, INF-*α*2, IL-12p70, GM-CSF, EGF, and VEGF measured in supernatants harvested from all different polarizing treatments and controls and analyzed in the Luminex MAGPIX® multiplexing instrument. For THP-1, mock: no stimulated cells; PMA: PMA-only control; M1: PMA + LPS + INF-*γ*; M2: PMA + IL-4 + IL-13; and M2 w/o PMA: IL-4 + IL-13. For U937, mock: no stimulated cells; PMA: PMA-only control; M1: PMA + LPS + INF-*γ*; M2-A: PMA + IL-4 + IL-13; M2-B: PMA + M-CSF; and M2-C: IL-4 + IL-13. For primary monocytes, mock: no stimulated cells; M1: GM-CSF + LPS + INF-*γ*; and M2: M-CSF + IL-4 + IL-13. U937 cells did not produce IL-12p70 under any condition. Asterisks and bars denote statistical significance between conditions. Significance ≤0.05 was indicated with *∗*, ≤0.01 was indicated with *∗∗*, and ≤0.0005 was indicated with *∗∗∗*. In THP-1 cells IL-1*β*, IL-8, G-CSF, and GM-CSF were significantly different between M1 versus M2 and M2 without PMA; EGF, (mock, PMA, and M1) versus M2 and M2 without PMA. In U937 cells IL-1*β*, G-CSF, INF-*α*2, and GM-CSF, M1 versus (M2-A, M2-B, and M2-C); EGF, PMA and M1 versus M2-A and M2-C. In primary monocytes IL-1*β*, IL-8, RANTES, G-CSF, INF-*α*2, IL-12p70, and GM-CSF, M1 versus M2; EGF, mock and M1 versus M2.

To fulfill their specific immune effector or remodeling functions, M1 and M2 macrophages should express a battery of specific cytokines, chemokines, and growth factors. To determine whether* in vitro* M1- and M2-polarized monocytes exhibited a specific profile of secreted immune modulators, we analyzed the supernatants of cells after the different treatments (Figure 5). Using a multiplex platform we tested cells for their capability to secrete proinflammatory cytokines, chemokines, and growth factors. Remarkably, in spite of our previous analyses in which we could not assign a specific M1 or M2 phenotype to the* in vitro* polarized monocytes/macrophages, we found a clear expression profile greatly conserved among all cells tested. IL-1*β*, IL-8, RANTES, G-CSF, IFN-*α*2, GM-CSF, and IL-12p70 were significantly increased in M1 conditions at least by one of the monocytes, while EGF clearly identified monocytes differentiated in M2 conditions (Figure 5). M1-polarized THP-1 cells presented a 111-fold increase of IL-1*β* over the value of M2-A treated cells; for U937 this cytokine was significantly higher in M1-polarizing treatment than all three M2 conditions; however the greatest difference was a 1544.5-fold increase in M1 over M2-C conditions, whereas for primary monocytes the difference was 8.6-fold. The level of IL-8 in supernatants of THP-1 cells was 46.2-fold higher in M1 conditions than in M2 conditions; the same comparison in primary monocytes resulted in a 2.7-fold difference. G-CSF increased 52.4-fold in THP-1 cells, 5656.12-fold in U937 cells (the highest increase seen between M1 and M2-C treatments), and 3.8-fold in primary monocytes in M1 conditions in comparison to M2 conditions. In spite of these numbers in which transformed cell lines seem to be more reactive than primary monocytes, it does not seem that polarized monocytic cell lines are more responsive than polarized primary monocytes. Primary monocytes presented a 1296.4-fold increased expression of GM-CSF and this chemokine only increased 18.6-fold in THP-1 cells and 195-fold in U937 (the greatest difference). The levels of RANTES and IL-12p70 in M1 treated primary monocytes were 77.6-fold and 121.5-fold higher, respectively, than the level found in M2 treated cells, while no significant changes were observed in the monocytic cell lines. If concentrations are considered, the levels of IL-12p70 and GM-CSF are still higher in M1-polarized primary monocytes than in any of the monocytic cell lines. There were no significant changes in the levels of MCP-1 and VEGF mediated by any treatment condition.

### 3.6. Phenotypic Changes Induced by Breast Cancer Cell Lines

**Figure 6:**
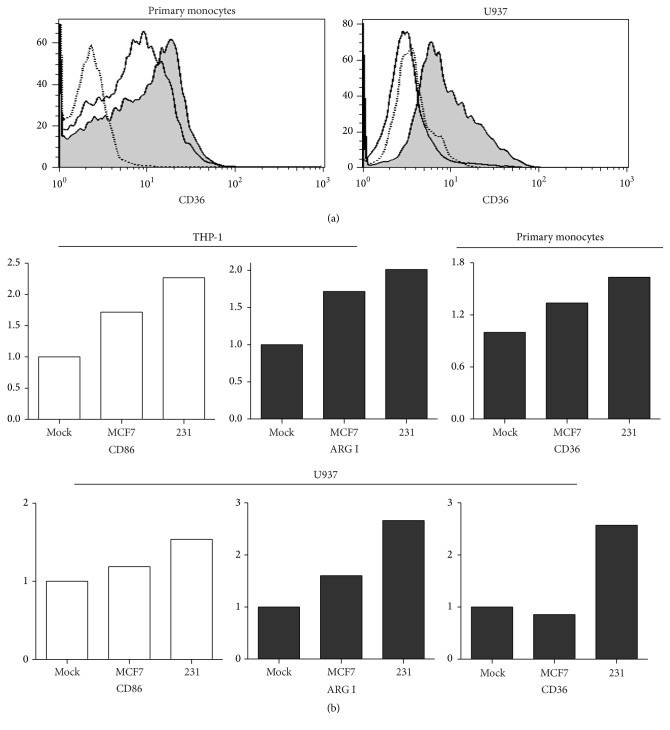
M1 and M2 polarization induced by breast cancer cell lines. After treatment of all three types of monocytes with either MCF-7 or MDA-MB-231 conditioned media, cells were harvested and the panel of M1/M2-related markers was analyzed by flow cytometry. In (a) the result for M2-related marker CD36 in primary monocytes and U937 cells is depicted: dotted line histograms represent autofluorescence control and straight line and shaded histograms correspond to stimulation with MCF-7 and MDA-MB-231 conditioned media, respectively. In (b) the autofluorescence was used to normalize marker expression, a value of 1 to the level of autofluorescence, and expression after treatment with conditioned media was normalized dividing by this basal value. Fold changes are depicted and the markers represented are only those with changes ≥ 1.5-fold. White bars represent M1 markers and black bars M2 markers.

We have previously documented that monocytes grown in the presence of conditioned media from breast cancer cell lines display characteristics of protumoral macrophages. Those monocytes expressed higher levels of COX-2, prostaglandin E2, and metalloproteinases and exhibited an increased extracellular matrix (ECM) remodeling activity [19]. Interestingly, invasive/metastatic breast cancer cells better promoted those changes than noninvasive cells [19]. We hypothesized that engaged monocytes to perform protumoral functions would better correlate with an M2 phenotype. To address that, we cultured the monocytic cell lines and primary monocytes for five days with conditioned media from MCF-7 and MDA-MB-231 breast cancer cell lines and analyzed M1/M2-related markers by flow cytometry. MCF-7 cells have been characterized as poorly aggressive (low invasive and nonmetastatic potential) tumor cells while MDA-MB-231 cells are well known as being highly aggressive because of their invasive and metastatic capacity. The result is shown in Figure 6; first with histograms of CD36 as an example, both primary monocytes and U937 cells exhibited changes in this marker especially after treatment with MDA-MB-231 conditioned media (Figure 6(a)). In Figure 6(b) fold changes are depicted; here basal expression of the marker in mock treated cells was given a value of 1, and expression after treatment with conditioned media was normalized dividing by this basal value. Only changes > 1.5-fold are presented; white bars represent M1 markers and black bars M2 markers. In general, we found that the aggressive MDA-MB-231 cell line induced more dramatic changes than the noninvasive cell line in all types of monocytes tested. Interestingly, those changes were not exclusive of M2 markers as we would expect considering the protumor activity conferred to the monocytes after treatment. THP-1 cells showed increased expression of M2 marker arginase I but also of M1 marker CD86. Primary monocytes only changed the expression of the M2 marker CD36. U937 cells changed M1 marker CD86 and M2 markers arginase I and CD36. These results reflect the ambiguity of phenotypes based on M1/M2 marker panels and suggest that protumoral macrophages cannot be defined as M1 or M2 based on these markers.

## 4. Discussion

Monocytes are highly reactive cells that undergo morphological, phenotypic, and functional changes when exposed to different stimuli. Although it is relatively easy to experimentally induce macrophages to perform specific activating functions, to assign a reliable phenotypic stage matching the plethora of functions that they perform remains a challenge. Up to today, the ideal technical guidelines to generate desired specific macrophage phenotypes have not been achieved due to the heterogeneity in reported experimental procedures and phenotypic markers [32]. We have previously developed experimental conditions in which monocytes can be coaxed to perform protumoral activities [19]. Since TAMs have been thought to perform protumoral activities that better correlate with M2 macrophage functions, we assumed that our experimental conditions would generate M2 phenotypes. However, when we analyzed three sources of monocytes, two cell lines derived from cancer patients and peripheral blood primary monocytes derived from healthy donors, which were subjected to a series of stimuli extensively reported in the literature, plus conditioned media from aggressive breast cancer cells, we found no clear phenotype correlating with M1 or M2 macrophages but a mix of marker expressions.

In this study, we first established the baseline identity of the monocytes confirming that they were undifferentiated monocytes with the classical associated morphology. When these cells were subjected to different stimulating conditions, cells underwent morphological changes that indicated their response to the stimulus; among these changes adherence is considered a feature of differentiation, signaling that monocytes have matured into macrophages, probably reflecting tissue resident macrophages. We then analyzed two panels of markers related to M1 and M2 macrophage programs, according to previous reports [1, 18, 33–35]. M1-related markers were CD86, TNF-*α*, CCR7, and IDO enzyme. Based on our results, while IDO enzyme and CD86 were significantly associated with M1 treatment in primary monocytes, TNF-*α* and CCR7 were not associated with any particular condition. When M2 selected markers (CD163, arginase I enzyme, CD36, and IL-10) were analyzed, data were even less correlative with M2 polarization conditions.

It is important to state that if we had only compared a specific monocyte polarizing condition against mock treated cells we would have obtained significant and correlative upregulation of the M1 or M2 markers. It is because we decided to be more rigorous, comparing marker expressions between M1- and M2-polarized cells, that no significant results were obtained for most markers. Our data support that M1 and M2 markers are upregulated by both polarizing conditions and also by protumoral stimuli. Of interest too is the fact that basal expression levels of these markers were very variable between monocyte types, in spite of a highly similar starting differentiation stage. We also observed a high level of variation within the same type of monocyte. We confirmed that variability after multiple series of experiments, in which two conditions were compared first, and then more polarizing conditions were added, plus PMA alone to address whether PMA was a major trigger of M1/M2 marker expression and thus responsible for the ambiguity of the data. We believe that marker upregulation reflects very reactive cellular stages already observed at basal culture conditions. Still, M1- or M2-polarizing conditions resulted in a more dramatic upregulation of marker expression.

Recent data support the notion that tumor progression results not only from genetic changes in the tumor cell itself, but also from the communication that it establishes with surrounding cells. The inflammatory microenvironment in which the tumor develops has been found to be critical for tumor initiation and growth [36]. In breast cancer, macrophages are particularly enriched at the invasive front and in the vascular areas of the tumor, possibly facilitating tumor invasion and metastasis [37]. Because M2 macrophages participate in tissue maintenance, increasing cell survival and proliferation and tissue angiogenesis, they are thought responsible for those protumoral activities [38]. The above observations support a model in which the tumor microenvironment actively recruits peripheral monocytes promoting their polarization into M2 macrophages and thus coaxing them to perform functions more attuned to the tumor needs [16]. In agreement, a meta-analysis showed that in >80% of breast cancer patients an elevated tumor macrophage density correlates with poor prognosis [39].

It is presently unclear how tumor and macrophages communicate to establish the tumor-promoting conditions. Inflammatory mediators and inflammatory targets with protumor activities have been described. It is thought that protumoral macrophages favor tumor growth through secretion of interleukins, chemokines, growth factors, angiogenic factors, proteases, and immunomodulatory molecules [40, 41]. We have previously observed that an aggressive breast cancer cell line engaged monocytes to express inflammatory mediators COX-2 and prostaglandin E2 and metalloproteinases, which rendered tumor and monocyte cocultures with an increased proteolytic capacity of ECM components. In this study, we found that M1-polarizing conditions result in higher expression/secretion of inflammatory mediators than M2-polarizing conditions. Only EGF secretion was found favored in M2-polarizing conditions, while IL-1*β*, IL-8, RANTES, G-CSF, MCP-1, IFN-*α*2, IL-12p70, and GM-CSF were all favored in M1 conditions. This result agrees that chronic stimulation with LPS and IFN-*γ* is more suitable to establish highly inflammatory stages than those promoted by IL-4 and IL-13 or that monocytes are more reactive to them. It is easy to envision how this inflammatory admixture may contribute to tumor initiation and progression. For instance, clinical studies have found that RANTES promotes progression of the most aggressive triple negative breast cancers [42]. RANTES and MCP-1 belong to the same CC chemokine family and coexpression of RANTES and MCP-1 has been observed in advanced human breast cancers [43]. Concomitant expressions of RANTES and IL-1*β* have also been observed in breast cancer relapsing patients [44]. These data support important protumoral functions by this mix of inflammatory components arguing that M1-polarizing conditions can also be critical to shape a microenvironment supporting of tumor growth.

Interestingly, although we observed an increased expression/secretion of inflammatory mediators after monocytes were subjected to M1-polarizing conditions, the resulting polarized cells did not match an M1 or M2 exclusive phenotype. Moreover, conditioned media from tumor cells did not result in formation of macrophages with a clearly defined M2 phenotype, an experimental condition with which we have previously coaxed monocytes to perform protumoral activities. Contrary to many immune cells that are terminally differentiated through extensive epigenetic modifications, monocytes/macrophages are highly plastic cells that remain responsive to environmental signals even after polarization into a specific subtype [45]. Since Mills and colleagues pioneering studies [3], it was clear that the M1 and M2 classification was better helpful to explain different metabolic responses than effector functions. Mosser and Edwards [4] stated that macrophages may only exist in a spectrum of different phenotypes and functions in which perfectly separable M1 and M2 subtypes may only exist at the opposite ends. In agreement, monocyte polarization with a plethora of different signals generated macrophages with at least nine different transcriptional programs [46]. Moreover, TAMs isolated from the MMTV-PyMT murine breast cancer model did not show a transcriptional program related to either M1 or M2 subtypes, and instead a Notch signaling fingerprint was found [47]. Although the existence of protumoral macrophages is unquestionable, their associated phenotypes and the precise conditions driving their formation are still an area in need of extensive research. Importantly, our data support that the macrophage-tumor cooperation may need a mix of inflammatory and anti-inflammatory stimuli together with a mix of M1 and M2 activities, and this has critical implications in the M2-to-M1 reverse polarization as a therapeutic option.
